# Immunothrombosis in Acute Respiratory Dysfunction of COVID-19

**DOI:** 10.3389/fimmu.2021.651545

**Published:** 2021-06-02

**Authors:** Xiang-Zhi Fang, Ya-Xin Wang, Ji-Qain Xu, Ya-Jun He, Zhe-Kang Peng, You Shang

**Affiliations:** ^1^ Department of Critical Care Medicine, Union Hospital, Tongji Medical College, Huazhong University of Science and Technology, Wuhan, China; ^2^ Institute of Anesthesiology and Critical Care Medicine, Union Hospital, Tongji Medical College, Huazhong University of Science and Technology, Wuhan, China

**Keywords:** COVID-19, inflammation, thrombosis, respiratory dysfunction, therapeutic

## Abstract

COVID-19 is an acute, complex disorder that was caused by a new β-coronavirus severe acute respiratory syndrome coronavirus 2 (SARS-CoV-2). Based on current reports, it was surprising that the characteristics of many patients with COVID-19, who fulfil the Berlin criteria for acute respiratory distress syndrome (ARDS), are not always like those of patients with typical ARDS and can change over time. While the mechanisms of COVID-19–related respiratory dysfunction in COVID-19 have not yet been fully elucidated, pulmonary microvascular thrombosis is speculated to be involved. Considering that thrombosis is highly related to other inflammatory lung diseases, immunothrombosis, a two-way process that links coagulation and inflammation, seems to be involved in the pathophysiology of COVID-19, including respiratory dysfunction. Thus, the current manuscript will describe the proinflammatory milieu in COVID-19, summarize current evidence of thrombosis in COVID-19, and discuss possible interactions between these two.

## Introduction

The novel coronavirus disease 2019 (COVID-19) pandemic, which first broke out in Wuhan, China, has now grown into a global pandemic. COVID-19 is an acute, complex disorder that is caused by a new β-coronavirus severe acute respiratory syndrome coronavirus 2 (SARS-CoV-2) (1). Similar to other human coronaviruses, SARS-CoV-2 mainly affects the respiratory system. While most people with COVID-19 present with only mild illness (2), approximately 5% to 16% require intensive care (3–5), among whom 50% to 70% rapidly progress to severe fatal respiratory dysfunction requiring mechanical ventilation especially acute respiratory distress syndrome (ARDS) (5, 6). Elderly individuals and persons with comorbidities have higher mortality rates (7). Surprisingly, the characteristics of many patients with COVID-19, who fulfil the Berlin criteria for ARDS, are not always like those of patients with typical ARDS and can change over time (8). Of note, COVID-19 patients develop profound hypoxemia early in their disease course, characterized by better compliance of the respiratory system, a low ventilation-to-perfusion (VA/Q) ratio and low lung recruitability, which is termed L-type ARDS (9). The initial acute respiratory dysfunction of COVID-19 dominantly results from pulmonary microvascular thrombosis, which is supported by pathological reports (10).

Studies have revealed that COVID-19 often causes episodes of thrombosis, and the incidence is higher in severe cases (11, 12). Recently published studies have considered thrombosis an important factor contributing to COVID-19 pathogenesis, especially respiratory dysfunction (11, 13). Additionally, thrombosis appears to be associated with other inflammatory lung diseases, such as acute respiratory distress syndrome and influenza-associated pneumonia (14, 15). Interestingly, inflammation predisposes patients to thrombosis, and conversely, thrombosis is associated with inflammation, a process sometimes known as immunothrombosis (16).

In this review, we attempt to describe the proinflammatory milieu in COVID-19, summarize current evidence of thrombosis in COVID-19, and identify possible interactions between inflammation and thrombosis. Given the relative lack of information in relation to COVID-19 thrombosis, we also summarize information from other similar RNA viral infections and inflammatory disorders.

## The Proinflammatory Milieu In Covid-19

The pathogenesis of COVID-19 is associated with a hyperinflammatory response (17, 18). The first step in the pathogenesis of COVID-19 is SARS-CoV-2 entry into target cells through its S proteins outside the viral lipid layer (19, 20). After entering host cells, mainly respiratory epithelial cells, SARS-CoV-2–expressing pathogen-associated molecular pattern molecules (PAMPs) activate a large number of innate and adaptive immune cells (21, 22), leading to the production of inflammatory cytokines and the type I interferons (IFN) IFN-α and IFN-β, which establish a proinflammatory milieu.

### The Cytokine Storm in COVID-19

In 1993, Ferrara (23) first proposed the concept of a cytokine storm in acute graft-versus-host disease. Since then, this concept has been further extended to other diseases, such as rheumatological disease and sepsis. Macrophage activation syndrome (MAS) refers to the cytokine storm induced by autoimmune disorders (24). Additionally, the cytokine storm that occurs after chimeric antigen receptor (CAR) T cell therapy is called cytokine release syndrome (CRS) (25). Elevated serum levels of interleukin (IL)-6 IL-7, IL-2, granulocyte colony-stimulating factor (G-CSF), macrophage inflammatory protein 1α (MIP1α), and tumor necrosis factor-α (TNF-α) have been reported in COVID-19 patients (2, 26, 27), which was widely recognized as cytokine storm.

CD8^+^ and CD4^+^ T cells are the most dominant cells that participate in the immune responses to SARS-CoV-2 infection (28). Recently, a clinical experiment suggested that SARS-CoV-2–specific CD8^+^ and CD4^+^ T cells were present in ∼70% and 100% of COVID-19 convalescent patients, respectively (29). Other immunological cells involved in SARS-CoV-2 infection include B cells, macrophages, T helper (Th) cells, neutrophils, and natural killer/cytotoxic T lymphocytes (CTLs) (21, 30–32). Excessively secreted cytokines attract neutrophils, monocytes, and macrophages to the site of the insult, where they not only clear viral particles but also may cause organ failure (33). Importantly, these cytokines also activate immune cells, further increasing the production of cytokines.

While the specific details and mechanisms of the cytokine storm in COVID-19 remain unclear, researchers suppose that this cytokine storm is connected with a dysfunctional immune response to remove the virus (34, 35). The immune response to SARS-CoV-2 includes two different phases. The first phase is the incubation phase, in which recruited cells and released cytokines fight SARS-CoV-2 infection. In most individuals, the immune response clears SARS-CoV-2, the immune response recedes, and patients recover. However, when there is a failure to fight SARS-CoV-2, the immune response enters the second phase. During this phase, an overactive immune response occurs, compensating for the target clearance failure, whereby clinical manifestations of a cytokine storm are present. Additionally, it is noteworthy that a recent study showed that SARS-CoV-2 coding protein open reading frame 8 (ORF8) activates the IL-17 signaling pathway and promotes the cytokine storm during COVID-19 (36).

### Neutrophil Extracellular Traps in COVID-19

Neutrophils are the most abundant white blood cell type circulating in the human bloodstream (37). When a pathogen enters the body, neutrophils, as the key components of the innate immune cell population, are recruited to infection or inflammation sites, where they activate other types of immune cells and eliminate pathogens (38–40). In 2004, Brinkmann et al. (41) first observed that, in response to bacterial endotoxins and inflammatory cytokines or drugs, circulating neutrophils form web-like structures that are commonly referred to as neutrophil extracellular traps (NETs). These NETs are composed of intracellular components released by activated neutrophils, including neutrophil elastase (NE), myeloperoxidase (MPO), histones, defensins, calprotectin matrix metalloproteinase-9, and cathepsin G (42). As part of the innate immune system, the main function of NETs is to trap and possibly even kill microorganisms. However, a large number of studies have confirmed that the activities of NETs is a double-edged sword, because, in addition to their microbicidal activity, NETs have been implicated in various tissue damage and involved in the development of sepsis and influenza pneumonia (43, 44).

An autopsy specimen from a patient who has succumbed to COVID-19 showed neutrophil infiltration in lung tissues (45). Elevated number of peripheral blood neutrophils is considered an early indicator of SARS-CoV-2 infection, associating with severe respiratory dysfunction and worse clinical outcomes (46, 47). Recent study has demonstrated that NET markers including cell-free DNA, myeloperoxidase (MPO)-DNA, and citrullinated histone H3 (Cit-H3) were significantly increased in serum samples from severe COVID-19 patients (48). Other studies found that increased plasma NETs are positively correlated with COVID-19 severity (49, 50). Importantly, SARS-CoV-2 can directly lead to the formation of NETs in healthy neutrophils through ACE2–serine protease axis and PAD-4 signaling (51).

## Thehemostatic Abnormalities And Pulmonary Microvascular Thrombosis In Covid-19

Ebolavirus, dengue fever, and Lassa virus, similar to SARS-CoV-2 virus, are enveloped, single-stranded RNA viruses that are thought to promote thrombosis (52, 53). To date, the human coronavirus most closely related to SARS-CoV-2 is SARS-CoV-1 (54). SARS-CoV-1 infection has been associated with hematological abnormalities, including thrombocytosis (49%), elevated D-dimer (45.0%), thrombocytopenia (55%), and prolonged activated partial thromboplastin time (aPTT) (63%) (55, 56). Chong et al. also reported that 20.5% of patients infected with SARS-CoV-1 had deep vein thrombosis, and 11.4% of these patients showed clinical evidence of pulmonary embolism (57). Furthermore, in a SARS-CoV-1–infected patient, edema and fibrin thrombi were identified in the pulmonary vasculature (58). Additionally, postmortem examinations from patients infected with SARS-CoV-1 revealed that thrombi were present in pulmonary, bronchial, and small lung veins, which implied a prothrombotic effect in the pulmonary vasculature of SARS-CoV-1–infected patients (59–61).

In addition to systemic hyperinflammation, SARS-CoV-2 infection is associated with coagulation abnormalities, which cause hypoxemic respiratory failure. COVID-19 patients appear to be more susceptible to thrombotic complications. This blood coagulation seems to be not only faster, but also more severe than that observed in life-threatening influenza or sepsis. Purple rashes, swollen legs, and clogged catheters are common clinical manifestations of COVID-19 (62, 63). A study from Wuhan China of critical COVID-19 pneumonia documented acro-ischemia including dry gangrene finger/toe cyanosis, and skin bulla, which accounted for 21% of critically ill patients hospitalized at the same time (62, 64). Recently, Helms and colleagues reported that more pulmonary embolisms were diagnosed in COVID-19 ARDS patients than in patients with non-COVID-19 ARDS (65). Despite widespread use of thromboprophylaxis, incidences of pulmonary embolism have still been reported to be as high as 21% in COVID-19 (66), which was two-fold higher than that in critically ill influenza patients. Importantly, pathological examinations revealed marked lung microvascular congestion in patients who died in early stages of COVID-19 (67).

According to different reports (12, 68, 69), the most common hematological disorder in COVID-19 patients is consistently represented by elevations in D-dimer and prothrombin time, and a relatively modest decrease in platelet count. Of particular note, increased D-dimer concentration was associated with a higher COVID-19 mortality rate. Additionally, the laboratory parameters in COVID-19 patients are distinct from those in patients with sepsis-induced coagulopathy and disseminated intravascular coagulation (DIC), who present with more serious thrombocytopenia and slight D-dimer abnormalities than COVID-19 patients (70). Likewise, COVID-19 patients are predominated by hypercoagulable state, with only 2% to 3% of patients presenting with serious bleeding (65). Collectively, this available evidence implies that hemostatic abnormalities and pulmonary intravascular coagulopathy occur in patients with COVID-19, even in the early course of the disease.

## Links Between Inflammation And Thrombosis In Covid-19

**Figure 1:**
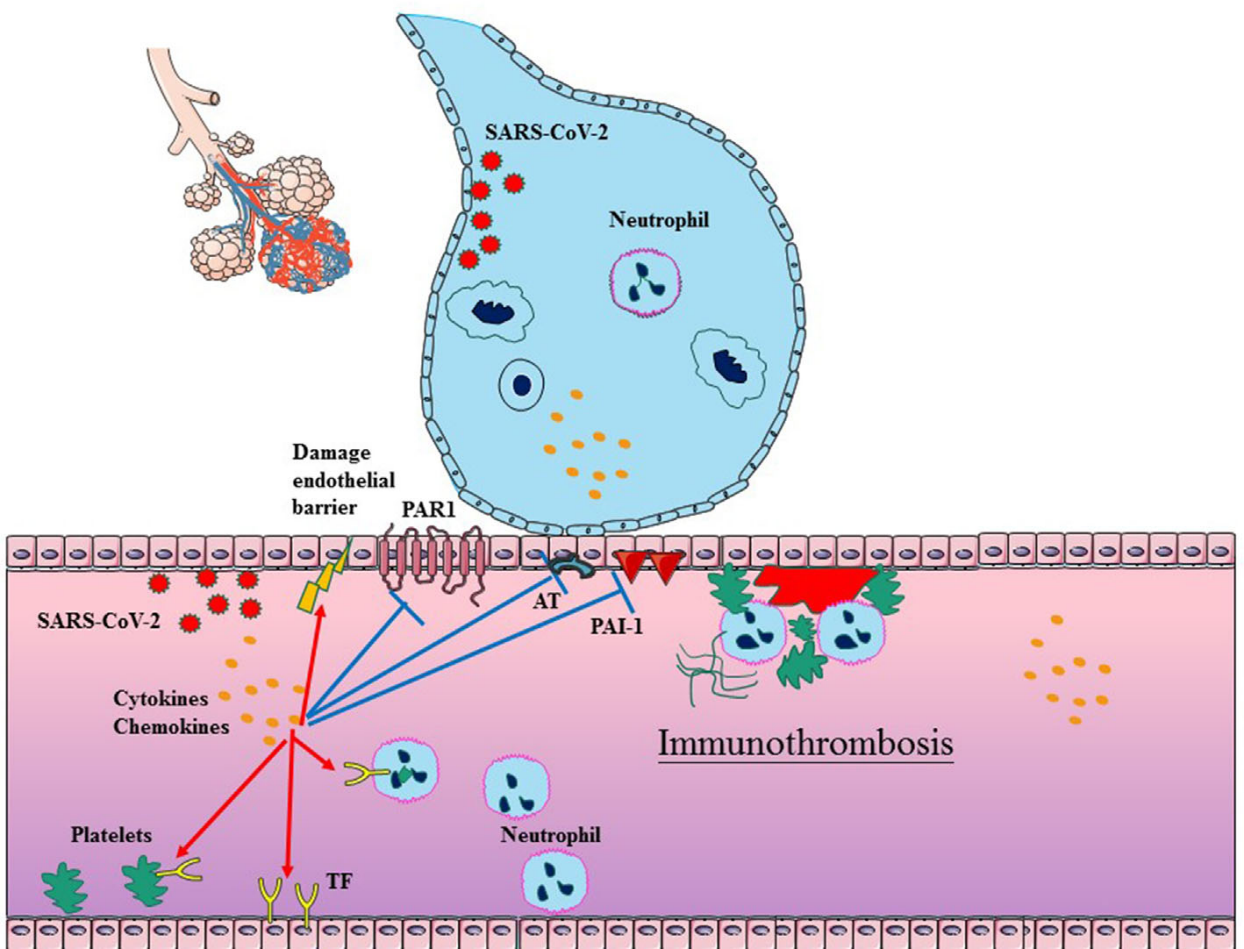
Schematic diagram of immunothrombosis in COVID-19. SARS-CoV-2 infection typically begins in epithelial cells of the respiratory tract, which can further promote the cytokine storm and infiltration of neutrophil. The cytokine storm can result in endothelial dysfunction, tissue factor (TF) overexpression, and the platelets activation, which initiates the TF-dependent coagulation process. The release of NETs promotes platelet–neutrophil aggregation and neutrophil activation in the bloodstream. Furthermore, the cytokine storm also suppresses antithrombin, APC and PAI-1, which impair anticoagulation and decrease fibrinolysis. In addition, SARS-CoV-2 can also directly impair ECs. The above factors collectively facilitate the generation of thrombus in COVID-19.

It has been shown that the immune response actively participates in the formation of thrombi within blood vessels, particularly in microvessels (71–73). The process, defined as immunothrombosis, accurately describes the intricate network between the coagulation system and the innate immune system (74). Immunothrombosis can locally confine an infection by facilitating the recognition, containment, and destruction of pathogens. Considering that the similar mechanisms exist in COVID-19 and bacterial sepsis, immunothrombosis has been reconsidered in SARS-CoV-2 infection. There are complex interactions between inflammation and thrombosis, involving endothelial cells (ECs), coagulation (activated TF, platelets, and neutrophils), anticoagulation (impaired AT, APC and TFPI systems), and decreased fibrinolysis (**Figure 1**).

### Endotheliitis and Endothelial Dysfunction

Endothelial cells (ECs) maintain the balance between the coagulation and anticoagulation systems of blood by expressing several mediators that prevent platelet activation and suppress coagulation and thrombus formation (75). Under normal conditions, ECs provide an environment with a slight tendency to promote anticoagulation in blood vessels (76). Therefore, dysfunctional ECs may contribute to the pathogenesis of thrombosis by altering the expression of pro- and antithrombotic factors.

Both autopsy findings and clinical observations have described the coexistence of vascular damage and thrombotic complications in a wide range of organs (77, 78), which provides indirect evidence to support that endotheliitis has an important role in immunothrombosis processes. In addition, COVID-19 patients have a higher circulating endothelial cell (CEC) count, suggesting pronounced endothelial injury in COVID-19 (79). A single-center study found that endotheliopathy might be an important factor in the pathophysiology of COVID-19–associated coagulopathy (80).

Transmission electron microscopy of the endothelium in COVID-19 showed ultrastructural damage, as well as the presence of intracellular and extracellular SARS-CoV-2 (81, 82). Perivascular inflammatory cell infiltration was observed in COVID-19 patients (82, 83). Thus, endotheliitis occurs in several organs, especially in the lung, as a consequence of direct SARS-CoV-2 infection and an overactive host immune response. Among the various inflammatory cytokines, IL-6 is one of the most major cytokines involved in cytokine storms in COVID-19 patients. L-6 has been confirmed to activate ECs, thus resulting in endothelial dysfunction, further activation of the coagulation cascades (84). Further larger studies are required to provide more direct evidence for the link between cytokine storm and thrombus formation in COVID-19.

### Tissue Factor

As the key element in the initiation of the extrinsic pathway of the blood coagulation cascade, tissue factor (TF) is constitutively expressed in perivascular cells, including adventitial fibroblasts, pericytes, and epithelial cells of the lung, brain, skin and kidney (85–87). Interestingly, vascular cells in contact with the blood, such as smooth muscle cells (SMCs), endothelial cells (ECs), alveolar epithelial cells, macrophages and neutrophils, do not express considerable amounts of TF in a quiescent state but can be transiently induced to do so under inflammatory conditions (88, 89). TF is encrypted under normal physiological conditions (90). In response to injury and inflammation, TF in vascular endothelial cells is released into the bloodstream and becomes exposed to circulating FVII/FVIIa. After the binding and activation of FVII, the TF-activated VII (FVIIa) complex consequently activates FX to FXa, which then interacts with its cofactor, factor FVa, phospholipids, and calcium to constitute a prothrombinase complex (91, 92). The prothrombinase complex subsequently converts prothrombin into proteolytically active thrombin, leading to fibrin deposition and microthrombi formation. Although microthrombi act as a barrier against the invasion of pathogens in the initial defense procedure, they recruit and activate innate immune cells that generate the inflammatory response and further enhance TF expression (93). Xue et al. (94) reported that the increased plasma levels of TF are associated with the outcome in sepsis-induced ARDS. Additionally, blocking TF activity dramatically reduces physiological and histological lung injury and improves survival (95).

Three recent reviews speculate that TF may be involved in COVID-19–related thrombosis (96–98). Furthermore, Hotz and colleagues found increased expression of TF in monocytes in critically ill COVID-19 patients (99). Higher levels of TF^+^ platelets, TF^+^ granulocytes, and TF^+^ procoagulant microvesicles (MVs) are reported in COVID-19 patients (100). The active form of TF can disseminate around the body through the extracellular vesicles (EVs), which participate in sepsis-induced coagulopathy and thrombus formation (101, 102). Recent study has shown that EV-TF activity increased in COVID-19 patients, associating with elevated thrombotic risk (103). However, another study found that TF levels were not increased in BALF from COVID-19 patients (104). This contradictory result may be due to differences in sample and detection methods. These studies indicate that TF appears to be a critical determinant of the inflammatory thrombosis process in COVID-19. However, direct evidence that the proinflammatory milieu of COVID-19 induces TF overexpression is still lacking.

### Neutrophils and NETs

Neutrophils contribute to immunothrombosis depending on the formation of NETs. NETs were originally observed in patients’ thrombi (105). Researchers have gradually realized that NETs provide a scaffold to recruit red blood cells, platelets, and white blood cells and to bind plasma proteins (106). Ischemic stroke thrombi were found to be positive for DNA, MPO, and citrullinated histone H4 upon triple containment, and NETs were abundant in different types of stroke thrombi (107). Perhaps NETs facilitate the development of venous thrombosis because NETs can directly result in fiber remodeling. Moreover, intravascular thrombosis induced by NETs led to extensive microvascular obliteration and multiple organ failure in sepsis (108).

NETs have been shown to colocalize with thrombi in COVID-19 (49). In addition, a recent study demonstrated a strong correlation between markedly elevated NETs and D-dimer levels, a degradation product of fibrin (109). Interestingly, serum samples from patients with progressive/severe COVID-19 cause abundant formation of NETs in healthy donors, which suggests that patients with COVID-19 have circulating factors that induce NETs (110). Dismantling or inhibiting NETs in COVID-19 could ameliorate thrombotic tissue damage associated with ARDS and higher mortality rates (45). These data suggest a clinical link between NET formation and immunothrombosis, which may thereby contribute to the COVID-19 prothrombotic state.

### Platelets

The adhesion and activation of platelets are essential events of pathological thrombosis and inflammation during ARDS pathogenesis, as they participate in not only hemostasis but also the infectious response. During endothelial damage, platelets adhere and aggregate to the site of vascular injury *via* subendothelial collagen and von Willebrand factor (VWF) bound to glycoprotein receptors (GPs), such as GPVI, GPIa/IIa, and GPIb/IX/V, on the platelet surface (111). This initial platelet adhesion to the subendothelial matrix at the site of vascular injury promotes a series of downstream signaling responses, which switch platelets from an inactivated to an activated state. The change in platelet shape and platelet dense granule secretion is a characteristic feature of the activated state of platelets (112, 113). Equally notable is that dense granules are composed of multiple cytokines and other bioactive molecules, which are critical mediators in the complex microenvironment of blood coagulation but also take part in the inflammatory reaction process (114). In addition, platelets and their released products facilitate neutrophil aggregation and activation in damaged endothelial cells (115–117)

In patients with severe COVID-19, the activation of platelets during SARS-CoV-2 infection has been observed (99, 100, 118). A temporal trend of dropping platelet counts in COVID-19 patients could suggest a worsening thrombotic state, while an increase in platelet count was associated with improved survival and reduced thrombotic risk in COVID-19 (119). Furthermore, plasma VWF protein levels are consistently elevated in patients with severe COVID-19 and correlate with adverse outcomes, suggesting enhanced adhesive interactions between circulating platelets and the damaged vessel wall (120). Interestingly, platelets incubated in plasma from COVID-19 induced platelet activation. These data indicate that the inflammatory milieu is at least in part accountable for increased platelet activation in severe COVID-19.

### Antithrombin

Antithrombin (AT) is a plasma glycoprotein produced mainly by the liver, and AT has anticoagulant characteristics. The anticoagulant effect of AT is achieved through the inhibition of thrombin, plasmin, FIXa, Xa, XIa, and XII (121). Free plasma AT neutralizes coagulation enzymes in a slow, progressive manner since binding is very slow. When AT bind to heparin sulfate molecules on the vascular endothelial surface, a conformational change occurs to results in a ≥1,000-fold enhancement of AT activity. This suggests that heparin may be ineffective in patients with low AT levels. It has already been shown that AT induces endothelial cell release of prostacyclin, a molecule that prohibits platelet aggregation and activation and neutrephil infiltration (122). Furthermore, AT can interact directly with leukocytes and lymphocytes, inhibiting their interaction with endothelial cells and alleviating the severity of capillary leakage and subsequent organ damage (123).

Hyperinflammation can markedly decrease AT levels and glycosaminoglycan synthesis, which is associated with coagulation (124). Additionally, several recent studies have shown that plasma antithrombin values are significantly decreased in COVID-19, which is strongly associated with mortality in COVID-19 (125–127). At supplementation, using fresh frozen plasma (FFP) in COVID-19 patients may improve thrombosis prophylaxis and thus have an impact on their survival (126). Together, the results of these studies imply that AT may be the link between inflammation and thrombosis in COVID-19. However, further studies are needed to clarify the anti-inflammatory properties of AT in COVID-19.

### Activated Protein C

Protein C is a plasma serine protease zymogen with 419 amino acids. Upon thrombin is bound to thrombomodulin on the vascular endothelial surface, protein C is converted to activated protein C (APC), which exerts potent anticoagulant activity through its irreversible proteolytic inactivation of activated FV and neutralizes PAI-1 (128). The cleavage of activated FVIII is also strengthened by APC (129). Changes in levels of APC or protein C (PC) are associated with modifications in the risk of thrombosis. In addition to its anticoagulant activity, APC reduces the inflammatory response, inhibits apoptosis and protects the endothelial cell barrier. APC not only suppresses proinflammatory and proapoptotic signals but also enhances anti-inflammatory and antiapoptotic pathways (130, 131).

Since APC plays an essential role in coagulation and the immune response, it is possible that APC is involved in immunothrombosis in COVID-19 (132, 133). This concept is further supported by a trial study to derive and validate a predictive score for disease worsening in patients with COVID-19 (134). COVID-19 patients admitted to the intensive care unit (ICU) had lower levels of antithrombin activity and protein C activity as well as higher D-dimer and fibrinogen levels than COVID-19 patients admitted to a conventional ward. It is noteworthy that multivariate analysis identified decreased activity of protein C as significant predictors of worsening disease.

### Decreased Fibrinolysis

The fibrinolytic system, controlled by coagulation itself, removes fibrin from the vascular system, preventing increased amounts of clots in the microcirculation. Plasminogen is converted to plasmin by the action of urokinase (u-PA) and tissue plasminogen activator (t-PA), which is the central link in the fibrinolytic system. Therefore, PAI-1, a rapidly acting inhibitor of t-PA and u-PA is the main inhibitor of fibrinolysis (135).

When the level of PAI-1 in the circulation is elevated, fibrinolysis is impeded by inhibitory function of t-PA and u-PA, leading to failed removal of thrombi from the vascular wall. Binding PAI-1 to t-PA or u-PA forms an inactive complex, thus negatively mediating fibrinolysis in the vascular wall. It has been shown that fibrinolysis is impaired in sepsis, primarily due to an exaggerated release of PAI-1 as a result of endothelial dysfunction, given the coexistence of an inflammatory response and endothelial dysfunction (136). In addition, increased activated platelets may also release large amounts of PAI-1, as platelets are the major circulating pool of PAI-1 that can contribute to a high local concentration of PAI-1 at the site of a growing fibrin clot (137).

Impaired fibrinolysis has been suggested in COVID-19 patients, which could further heighten their thrombotic risk. This has been evidenced by markedly reduced clot lysis at 30 min *via* thromboelastography (TEG) in COVID-19 patients (138). Elevated levels of t-PA and PAI-1 were observed in patients during COVID-19, further suggesting impaired fibrinolytic ability (139, 140). IL-6 is the most clinically suitable biomarker for COVID-19. The latest research (141) found that the blockade of IL-6 signaling using tocilizumab treatment significantly decreases serum PAI-1 levels in patients with severe COVID-19.

## Summary And Conclusions

Emerging evidence has suggested that COVID-19 patients suffer from pulmonary microvascular thrombosis, which may explain the rapid deterioration and pulmonary collapse that is observed in patients who suddenly progress to ARDS. Immunothrombosis may be a key link between COVID-19 and thrombosis. The process involves a highly coordinated and mutual regulating process of joint participation of multiple factors, such as inflammatory cells, TF, endothelial dysfunction, and platelets. However, the pathophysiology of COVID-19 and thrombotic complications is complex, and the roles of many of the important factors, such as tissue factor pathway inhibitor and protease-activated receptors, are not fully understood. Accordingly, much attention should be directed to a deeper understanding of the pathogenesis of COVID-19.

## Data Availability Statement

The original contributions presented in the study are included in the article/supplementary material. Further inquiries can be directed to the corresponding author.

## Author Contributions

X-ZF and YS conceptualized and wrote the original draft of the manuscript. J-QX and Y-XW drew diagrams. Z-KP and Y-JH reviewed and edited the manuscript. All authors contributed to the article and approved the submitted version.

## Conflict of Interest

The authors declare that the research was conducted in the absence of any commercial or financial relationships that could be construed as a potential conflict of interest.
